# Configurational impact of self-regulated writing strategy, writing anxiety, and perceived writing difficulty on EFL writing performance: an fsQCA approach

**DOI:** 10.1038/s41598-024-61537-x

**Published:** 2024-05-15

**Authors:** Cunying Fan, Juan Wang

**Affiliations:** Department of College English Teaching, Qufu Normal University, Qufu, 273165 Shandong China

**Keywords:** Self-regulated writing strategies, Writing anxiety, Perceived writing difficulty, Writing performance, fsQCA, Psychology, Risk factors

## Abstract

Previous research has indicated that writing performance of foreign/second language (L2) learners is influenced by their utilization of self-regulated writing strategies. Yet, the relationship between various self-regulated strategies and individual characteristics, such as writing anxiety and perceived writing difficulty, has not been sufficiently examined. To bridge this gap, this study classified self-regulated writing strategies into four distinct types: cognitive, metacognitive, social behavioral, and motivational. These types were combined with L2 learners’ writing anxiety and writing difficulty to form conceptual models to predict high or low writing performance. Fuzzy-set qualitative comparative analysis (fsQCA) was used to gain a detailed understanding of the causal intricacies of writing performance. Data was collected from a sample of 94 students attending a university in eastern China. fsQCA revealed a variety of configurations associated with EFL writing performance, with six of them leading to high performance and four to low performance. These configurations highlight the complex causal relationship between students’ use of self-regulated writing strategies and their writing performance, while considering their writing anxiety and perceived writing difficulty. The study provided theoretical and practical implications for L2 teachers and educators who wish to enhance L2 learners’ writing performance.

## Introduction

In our globalized world, English’s role as the primary language for international communication has rendered English writing skills increasingly important, particularly in non-English-speaking countries like China. However, writing in English poses notable challenges for EFL learners^1^. These challenges arise partly because writing requires the recollection of information from memory, the organization of thoughts, the transformation of ideas into linguistic forms, the employment of writing instruments to articulate these ideas on paper, and the revision of the text to produce a polished and coherent final product^2^. Furthermore, research on Chinese university students shows that their performance in English writing is frequently hampered by encountered difficulties, experienced anxiety, and challenges in effectively regulating their writing^3^^,^^4^, underscoring the importance of a comprehensive understanding of the factors impacting their writing performance.

Self-regulated learning (SRL) involves individuals actively and deliberately taking charge of their learning processes. Self-regulated learners plan, monitor, and manage cognitive, motivational, emotional, and behavioral aspects of learning to gain knowledge and acquire skills^5^^,^^6^. Applying SRL to writing involves a strategic approach where learners proactively set writing goals, employ and monitor suitable writing strategies, and engage in reflective practices to evaluate and improve their writing. This method helps in effectively managing writing-related challenges such as anxiety and perceived difficulties, thereby enhancing overall writing performance. SRL in writing encapsulates a holistic process of planning, executing, and revising, fostering a more competent and confident approach to writing tasks.

Research has shown that self-regulated writing strategies can be beneficial in enhancing writing proficiency^7^^,^^8^. Students who implement these strategies tend to have better writing performance and higher self-efficacy^9^^,^^10^. Writing anxiety is an emotional, mental, or behavioural impediment to a writing task which leaners are cognitively capable of completing^11^. If learners suffer from writing anxiety, they may focus more on the details than the flow of the text, leading to mistakes and a lack of sincerity in the writing^12^ and are inversely related to writing performance^13^. Learners’ perception of writing difficulty is shaped by their proficiency and affective factors along with the complexity of the task they are assigned^14^. This is related to how much cognitive resources is required to successfully complete the task^15^, which ultimately affects their writing performance. Previous research has explored the individual effects of self-regulated writing strategies, writing anxiety, and writing difficulty on writing performance, but none have provided a comprehensive insight into the intricate relationship between them. This study employs fsQCA to investigate how students’ self-regulated writing strategies, along with the interrelated variables of writing anxiety and writing difficulty, collectively and configurationally influence their writing performance.

In this study, we utilize fsQCA, a configurational approach, to explore the complexities of the writing process. The term “configuration” here refers to the unique combination and interplay of various factors—self-regulated writing strategies, writing anxiety, writing difficulty, and EFL writing performance—within a complex system. Opting for fsQCA enables us to examine the non-linear interactions and cumulative effects of continuous variables^16^, thereby extending our analysis beyond conventional linear methodologies. Our goal with fsQCA is to reveal how different configurations of these factors influence EFL learners’ writing performance, offering an in-depth understanding of the intricate and multifaceted nature of the writing process.

This study enriches the EFL writing literature by utilizing fsQCA within the framework of self-regulated learning. It constructs and validates an intricate model to identify the determinants of high or low writing performance. This approach enables an in-depth examination of the interplay among key factors such as self-regulated writing strategies, writing anxiety, and writing difficulty. Our analysis of these factors’ configurations advances a non-linear and comprehensive understanding of writing performance. The insights gained from this study are invaluable, offering researchers and educators new perspectives to effectively tackle the diverse and complex challenges prevalent in EFL writing education.

## Literature review and research model

### Self-regulated learning

SRL is a process where individuals actively manage their own learning. This involves not only focusing on acquiring knowledge and skills but also managing cognitive, motivational, emotional, and behavioral aspects^5^^,^^6^. Self-regulated learners set goals, strategize, and reflect on their learning, believing that strategic application of these skills enhances academic achievement^17^. However, many students face challenges in effectively self-regulating their learning due to the complex and demanding nature of this process, potentially leading to cognitive overload^18^.

Incorporating SRL into the context of writing, particularly for addressing the challenges like writing anxiety, perceived writing difficulties, and writing performance, involves learners actively engaging in the SRL cycle to enhance their writing skills. This includes setting clear goals for writing tasks (forethought phase), employing and monitoring effective writing strategies (performance phase), and reflecting on the writing process to identify areas for improvement (self-reflection phase). By doing so, learners can manage their cognitive and emotional responses to writing tasks, reducing anxiety and perceived difficulties, and ultimately improving their overall writing performance. This proactive and strategic approach in the writing process exemplifies the essence of SRL in action, demonstrating its practical application in overcoming common writing challenges.

### Self-regulated writing strategy and writing performance

Self-regulated writing strategies have been found to be influential in writing proficiency and L2 writing quality^19^^,^^20^. Studies have shown that the implementation of such strategies can result in a marked improvement in writing proficiency^21^, particularly for those who struggle to acquire the necessary writing skills^22^. These strategies are essential in motivating, inspiring, and sustaining the dedication and perseverance of learners^23^^,^^24^, thus leading to better writing outcomes and improved writing performance^25^. Students who do not possess self-regulated writing strategies are more likely to experience negative emotions and be discouraged when confronted with writing tasks^20^^,^^26^^,^^27^. This can have a detrimental effect on their writing performance.

Teng and Zhang^8^ proposed a model for self-regulated strategies in L2 writing, comprised of cognitive, metacognitive, social-behavioral, and motivational regulation dimensions. Cognitive strategies refer to the techniques utilized by L2 writers to manage information processing while completing a task. Metacognitive strategies encompass the management of cognitive processes to make the most of cognitive resources and meet the requirements of the task. Social-behavioral strategies involve the efforts of L2 writers to adjust their learning behaviors in response to contextual and environmental factors. Finally, motivational regulation strategies denote the methods employed by L2 writers to sustain or increase their motivation, which can ultimately improve their engagement and success in completing the task. This study builds on Teng and Zhang’s^8^ model of self-regulated writing strategies, which acknowledges the multifaceted nature of self-regulated writing strategies^28^, and is specifically designed for Chinese university students, the same population this research is targeting. This study further investigates the various kinds of these strategies.

Research has demonstrated the impact of various self-regulated writing strategies on writing performance. Zimmerman^29^ emphasized the importance of emotional control strategies in managing negative emotions, such as anxiety or worries about writing. Bai et al.^22^ found that primary school students used various writing strategies, such as monitoring, evaluating, planning, resourcing, revising, and text-generating strategies, which were associated with their English competence. De Silva and Graham^9^ showed that proficiency in metacognitive writing strategies, including planning, monitoring, and evaluation, had a positive effect on writing outcomes. Qin and Zhang^30^ proposed that self-regulated writing strategies, such as evaluating, monitoring, and planning, were essential factors in predicting writing performance. Teng et al.^25^ revealed that in an EFL setting, writing performance of secondary school students is contingent on their understanding and application of writing strategies related to emotional control, goal-oriented evaluation, goal-oriented monitoring, memorization, metacognitive judgment, and planning.

However, while the benefits of self-regulated writing strategies are well-documented, the literature reveals a gap in understanding how these strategies interact with individual learner characteristics, such as writing anxiety and perceived writing difficulty. Most studies have focused on the strategies themselves, rather than how they combine with other factors to influence L2 writing performance. For instance, the model proposed by Teng and Zhang^8^ categorizes self-regulated strategies into cognitive, metacognitive, social-behavioral, and motivational dimensions but does not fully explore their interplay with individual psychological factors in an L2 writing context. The present study seeks to bridge this gap by examining the relationship between different types of self-regulated writing strategies, writing anxiety, and perceived writing difficulty, and their combined effect on L2 writing performance.

### Writing anxiety and writing performance

Anxiety related to writing can be a distinct form of anxiety^31^^,^^32^. Individuals who experience high levels of anxiety in writing tend to view writing as an unfulfilling task and it is possible for them to refrain from enrolling in writing classes and participating in situations where their written work will be assessed^33^. According to Cheng^31^, writing anxiety can be divided into three distinct categories: somatic, cognitive, and behavioral. Somatic anxiety is characterized by physical symptoms, such as a racing heart, gastrointestinal distress, and a feeling of tension. Cognitive anxiety is more psychological in nature, and involves worrying about one’s performance, having negative expectations, and being concerned about how others will view one's writing. Behavioral anxiety is demonstrated through avoidance, particularly in the form of avoiding writing. Cheng^31^ found that all three types of writing anxiety were negatively correlated with individuals’ enthusiasm for English writing courses, motivation to write in English, self-assurance in their English writing ability, and their performance on a timed English composition task.

Research has demonstrated that writing anxiety has a detrimental effect on writing performance^34^^,^^35^. Specifically, Zabihi^34^ found that writing anxiety had an adverse impact on the complexity, accuracy, and fluency of narrative performance, while Zabihi et al.^35^ found that it led to an increase in the number of errors present in students’ narratives. Furthermore, Abolhasani et al.^36^ found that undergraduates’ graph writing performance was impaired by their L2 writing anxiety. Conversely, writers with low levels of anxiety have been observed to exhibit fewer anxious writing behaviors, devote more time to ideation, produce multiple drafts, and allocate greater amounts of time to the writing process^37^.

Previous research has indicated a negative correlation between writing anxiety and writing performance, however, Lee^38^ conducted a study on Taiwanese EFL learners and found that writing anxiety did not have a significant effect on their writing performance. Payant et al.^39^ conducted another study that revealed writing anxiety to be a favorable predictor of performance on a source-based writing task. This was attributed to the fact that test anxiety, which had a beneficial effect on performance, was often experienced by participants. These conflicting results highlight the need for further investigation into how writing anxiety influences L2 writing performance.

### Perceived writing difficulty and writing performance

Writing difficulty perceived by L2 writers is a subjective judgement, which is significantly affected by their skill level and emotional state^14^.This perception is based on the allocation of cognitive resources or the mental effort required to fulfill the demands of the writing task^15^, which is a result of the interplay between personal endowments and features of writing tasks^40^. To gain a thorough comprehension of writing performance, it is essential to take into account writing difficulty perceived by L2 learners^14^, as this provides an essential explanation of the mental effort needed to handle cognitive loads in L2 writing.

Owing to the increased cognitive and linguistic demands involved in writing in a non-native language, a significant proportion of EFL learners perceive English writing as a difficult and challenging task. Rabab’ah^41^ observed that those who come from Asian universities often encountered difficulties when attempting to adjust to the requisites of English academic writing. The presence of negative thoughts about L2 writing or perceived difficulties regarding L2 writing had a significant impact on L2 writers’ ability to convey their ideas in writing^42^, thus influenced their writing performance. In addition to other factors, writing instructors concurred that students’ perception of difficult with EFL academic writing was a contributing factor to their poor writing performance^43^. Despited the negative influece of writing difficulty perception on writing performance, Wei and Zhang^44^ found that the degree of difficulty that L2 writers perceive in L2 writing could be indicative of their awareness of the inadequacy of their L2 writing knowledge or their uncertainty about the L2 writing process. This perception of writing difficutl could prompt Chinese EFL student writers to utilize their L1 rhetorical knowledge to aid in their L2 composing processes.

There has been a dearth of research examining the impact of perceived writing difficulty on the writing performance of L2 learners, let alone the interplay between this perception, self-regulated writing strategies, and writing anxiety. Consequently, this study examined these three factors and explored how they configurate to influence writing performance of L2 learners.

### Interplay of self-regulated writing strategy, writing anxiety, and perceived writing difficulty

In the realm of L2 writing research, the intricate interplay of strategy use, anxiety, and task difficulty has been explored to understand their collective impact on L2 writing performance. Zhou et al.^45^ used a structural equation modeling approach to investigate the relationships among L2 writing anxiety, L2 writing self-efficacy, L2 writing self-regulated strategies and L2 writing engagement, and possible mediators that regulate the effect of individual factors. A questionnaire was administered to 340 Chinese high school students and L2 writing anxiety was found negatively associated with L2 writing self-regulated strategies. Manson et al.^46^ discovered that the development of self-regulated strategies significantly and positively impacts students with learning disabilities (LD) across both elementary and secondary education levels. Notably, these strategies have been effective in reducing the students’ perceived writing difficulties.

### QCA in L2 writing

Understanding the application and importance of qualitative comparative analysis (QCA) in L2 writing research is crucial, given the complex nature of language learning and writing processes. QCA, as a method, stands out for its ability to handle complexity and multifaceted phenomena, which are inherent in L2 writing. This method is particularly suitable for analyzing L2 writing because it allows for the examination of various combinations of causal conditions (such as language proficiency, cognitive strategies, first language influence, and instructional methods) and their relationship to writing outcomes. Employing QCA, Mallahi et al.^47^ explored the role of a set of cognitive (i.e., aptitude and working memory) and motivational (i.e., self-regulatory capacity and self-efficacy beliefs) individual difference variables in the writing quality and composing behavior of 78 Iranian undergraduate EFL learners.

The applicability of QCA is further highlighted by its capacity to accommodate diverse data types and sources, making it well-suited for interdisciplinary research like L2 writing studies, which often integrate linguistic, psychological, and educational perspectives. Sazideh and Mallahi^48^ employed a qualitative case study approach, incorporating techniques like narrative construction and qualitative comparative analysis. They examined how individuals with diverse cognitive characteristics, including language learning aptitude and working memory, respond to various forms of feedback (e.g., direct, indirect with error codes, metalinguistic with explanations) on linguistic aspects of their writing. Additionally, they analyzed how these characteristics might impact their learning from the feedback, illustrating the influence of temporal and proficiency-related factors on the L2 writing process. This methodological versatility is essential for dissecting the layered dimensions of L2 writing, providing a more comprehensive understanding of how various factors interact to influence writing proficiency. Therefore, QCA emerges not just as a choice but as a necessary tool for researchers aiming to construct a holistic picture of L2 writing, accommodating its inherent complexity and the interplay of multiple influential factors.

A review of the literature has revealed a lack of understanding regarding the relationship between self-regulated writing strategies, writing anxiety, and writing difficulty and their impact on L2 writing performance. More precise and insightful outcomes can be obtained by taking into account the configurations of these factors. This study aims to answer the following question:

What configurations of self-regulated writing strategies, writing anxiety, and perceived writing difficulty, are associated with high and low writing performance in Chinese EFL learners?

### Conceptual model

**Figure 1:**
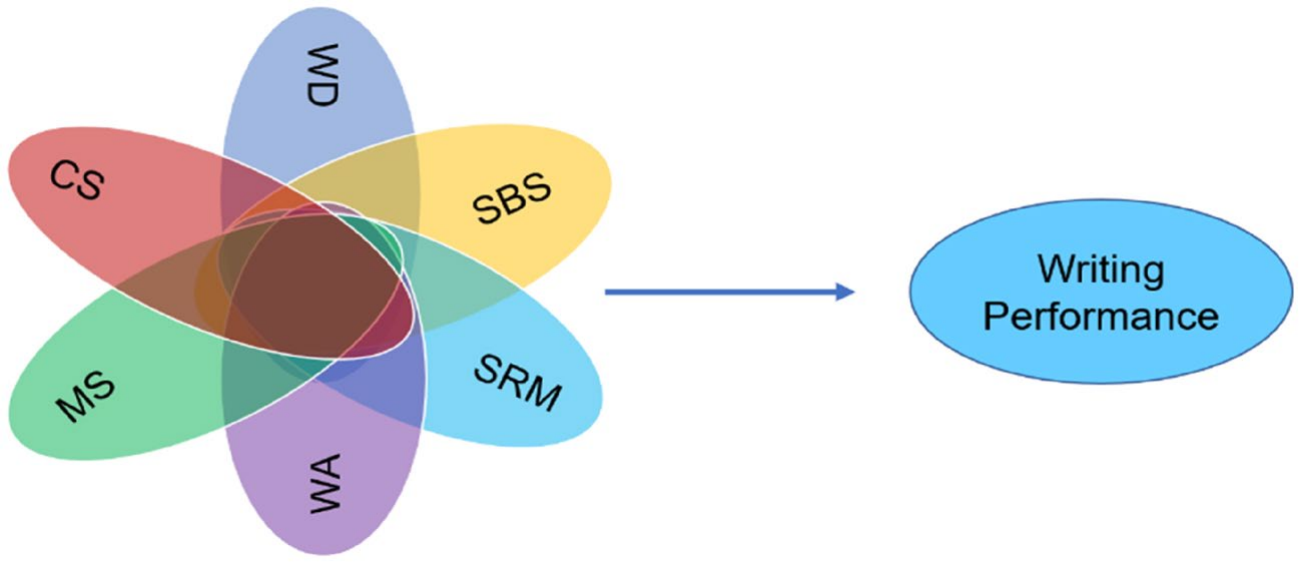
Venn diagram of the conceptual model. *CS* cognitive strategies, *MS* metacognitive strategies, *SBS* social-behavioral strategies, *MRS* motivational regulation strategies, *WA* writing anxiety, *WD* writing difficulty.

Research has indicated that self-regulated writing strategies are influential in determining L2 writing performance. However, the types of self-regulated writing strategies have not been fully explored. Moreover, the impact of such strategies may become complex when learners’ perception of writing anxiety and writing difficult is taken into consideration. Therefore, following Teng and Zhang^8^, we categorized writing strategies into cognitive strategies, metacognitive strategies, social-behavioral strategies, and motivational regulation strategies and integrated them with writing anxiety and writing difficulty to examine the interaction between these factors and their impact on writing performance. To investigate this relationship, we propose a conceptual model. Our model posits that EFL writing is a multifarious and intricate process, wherein writing performance can be accounted for by configuration of self-regulated writing strategies, writing anxiety, and perceived writing difficulty. Figure 1 showed our configurational research model.

## Method

### Fuzzy set qualitative comparative analysis

Qualitative comparative analysis (QCA) bridges the gap between qualitative and quantitative research^49^. It is a configurational approach that recognizes that social phenomena are often interconnected rather than isolated^16^. It comprises three modes of operation: crisp set QCA, multi-value set QCA, and fuzzy set QCA^50^. fsQCA is the chosen methodology for this study as it is especially suitable for dealing with issues that involve categorical variables as well as continuous variables^16^. It is useful to handle complex issues such as writing performance, which is affected by multiple factors.

The fsQCA is particularly effective for this analysis as it can explore how different combinations of variables contribute to writing performance, moving beyond the limitations of traditional statistical methods that typically focus on isolated impacts of individual variables. The use of fsQCA is advantageous in the context of language learning, which often involves complex, non-linear interactions among factors. This method allows us to identify specific configurations of factors that produce particular outcomes, providing a nuanced understanding of the collective impact of these elements on EFL writing performance. Our choice of fsQCA, especially considering our sample size of 94 participants, aligns with the recommendations of Poorkavoos et al.^51^. They noted the method’s suitability for small to medium-sized samples and its ability to uncover intricate causal relationships that might not be evident in larger datasets suited for regression analysis.

### Participants

A total of 107 undergraduate and postgraduate students from a university in Eastern China voluntarily participated in the 2022 FLTRP∙ETIC Cup English Writing Contest, a highly regarded annual national event. These participants were recruited through an open call for entries to all eligible students at the university, allowing any interested student to sign up freely. This process ensured that the selection of the 107 participants was random and voluntary, reflecting a diverse and representative sample. Out of these, 94 students completed a paper questionnaire that explored their use of self-regulated writing strategies, along with perceptions of writing anxiety and difficulty, resulting in an 87.85% response rate. The questionnaires were distributed following the contest, ensuring that the participants’ responses were based on their direct and recent experiences in the contest. This approach aimed to accurately capture and understand the self-regulation strategies and experiences of university students in English writing contexts.

**Figure 2:**
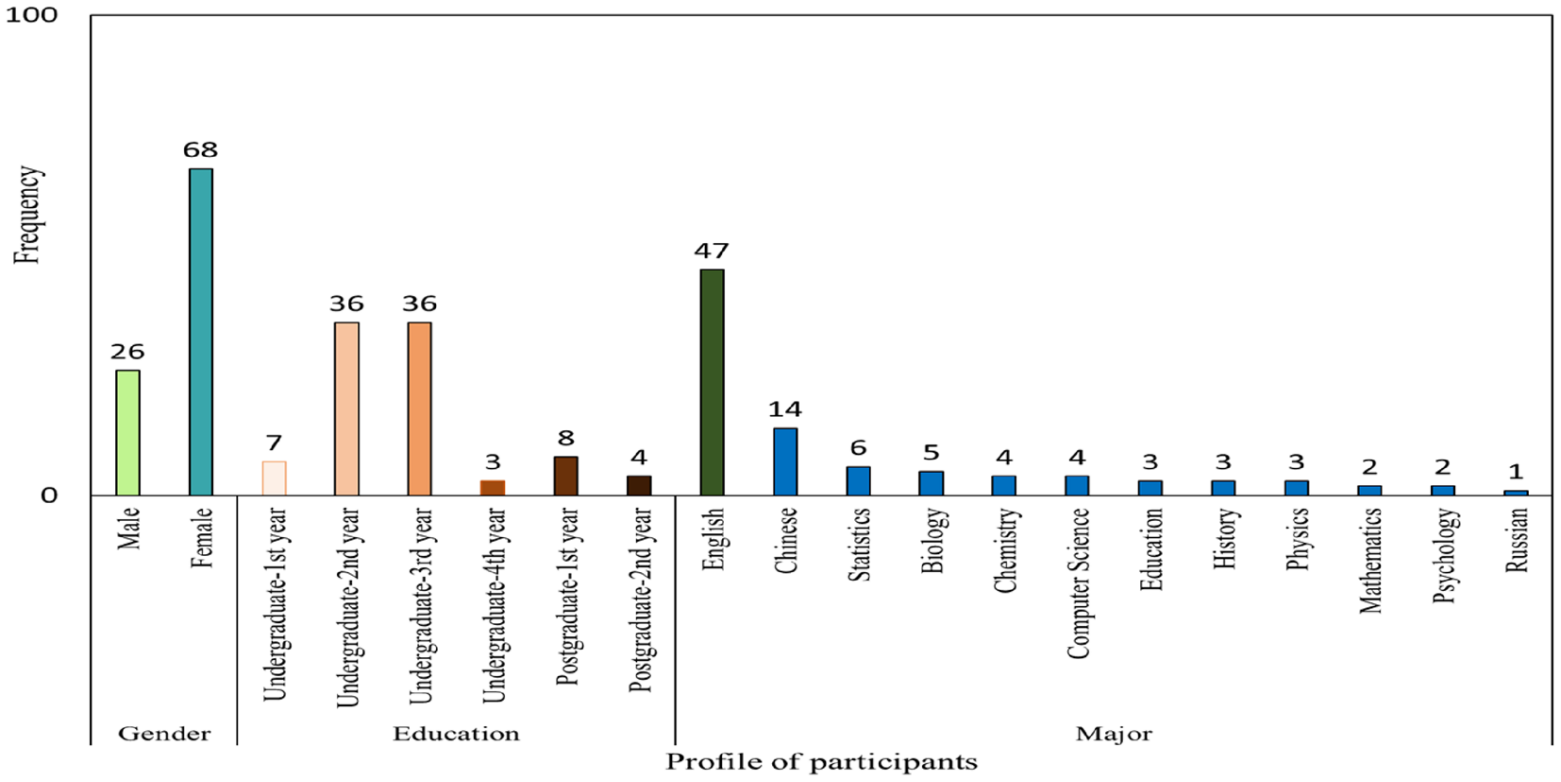
Demographic information of participants (n = 94).

Out of the 94 participants, 26 (27.66%) were male and 68 (72.34%) were female. Furthermore, 12 (12.77%) were postgraduates and 82 (87.23%) were undergraduates. 50% of the participants specialized in English, while the other half majored in non-English subjects. Figure 2 provides a visual representation of the participants’ demographic information.

### Measures

#### Writing performance

To accurately measure the writing performance of participants, we utilized the scores from the 2022 FLTRP∙ETIC Cup English Writing Contest, recognized as the most prestigious writing contest in China. The contest, spanning a duration of two hours, challenged participants in both argumentative and expository writing. It was evaluated on a total score of 100 points, with criteria based on comprehensive, rigorous, and equitable standards.

The judging criteria were detailed as follows: 40% on Content/Ideas, 30% on Organization/Development, 30% on Language. These criteria ensured a thorough assessment of participants’ ability to express clear ideas, organize content coherently, and use language effectively. Additionally, the contest utilized the iWrite English writing teaching and rating system for automated scoring support. This system, with a correction accuracy rate of 98% and a recall rate of 70%, ensures high consistency between human and machine ratings, with a consistency rate of 0.9. The system evaluates based on four dimensions: language, content, structure, and technical standards, offering customized scoring for different genres like application documents, argumentative essays, expository essays, narrative essays, and academic writing.

By employing these stringent and equitable criteria, along with advanced automated scoring technology, the contest provided an accurate assessment of participants’ writing performance, reflecting their skills in argumentative and expository writing.

#### Self-regulated writing strategies

The Writing Strategies for Self-Regulated Learning Questionnaire^8^, was used to evaluate the application of self-regulated writing strategies. This questionnaire comprised 40 items, focusing on dimensions of cognitive strategies, metacognitive strategies, social-behavioral strategies, and motivational regulation strategies. Each measured on a 7-point Liker scale, with scores ranging from 1 (not at all true of me) to 7 (very true of me).

Cognitive strategies (CS) refer to the strategies that students employ to process and utilize information or knowledge while completing a writing task. This dimension encompasses two aspects, namely text processing and course memory. The former, consisting of 6 items, assesses students’ utilization of linguistic, rhetorical, and discourse knowledge to produce a written text (e.g., When writing, I check the structure for logical coherence). The latter, comprising 3 items, evaluates students’ active retention of writing knowledge acquired from writing courses (e.g., I write useful words and expressions taught in writing courses to help me remember them).

Metacognitive strategies (MS) encompass three kinds of abilities that empower learners to manage and manipulate their own cognition and cognitive resources to fulfill the requirements of particular writing tasks. Idea planning, consisting of 3 items, refers to the specific behavior of generating ideas before writing (e.g., Before writing, I use the Internet to search for related information to help me plan). Goal-oriented monitoring and evaluating, consisting of 6 items, includes a range of strategies such as setting goals to direct writing activities (e.g., When learning to write, I set up goals for myself in order to direct my learning activities) and monitoring and evaluating knowledge and performance mastery in writing courses (e.g., I monitor my learning process in writing courses; I evaluate the mastery of the knowledge or skills learned in writing courses).

Social-behavioral strategies (SBS) involve conscious efforts by individuals to adjust their writing behavior in response to the context and environment. This dimension includes two main components: feedback handling and peer learning. The former, consisting of 4 items, relates to how students approach and react to feedback from both teachers and peers with the goal of enhancing their English writing abilities (e.g., I try to improve my English writing based on teachers’ feedback). The latter, comprising 3 items, involves seeking help from peers within the learning environment, thus constituting a social interaction (e.g., I discuss with my peers to have more ideas to write).

Motivational regulation strategies (MRS) are deliberate approaches used by students to maintain or enhance their motivation when engaging in writing tasks. This dimension encompasses motivational self-talk, interest enhancement, and emotional control. Motivational self-talk, consisting of 8 items, involves self-encouragement in knowledge mastery and academic performance (e.g., I remind myself about how important it is to get good grades in writing courses). Interest enhancement, comprising 4 items, reflects students’ inclination to make learning more enjoyable (e.g., I look for ways to bring more fun to the learning of writing). Emotional control, consisting of 3 items, measures learners’ efforts to minimize distractions when completing a writing task or learning to write (e.g., I find ways to regulate my mood when I want to give up writing).

The internal consistency of the dimensions of self-regulated strategies was found to be high, as evidenced by the reliability coefficients of 0.881, 0.891, 0.817, and 0.917, which surpass the accepted threshold of 0.7^52^^,^^53^, thereby demonstrating the questionnaire’s reliability.

#### Writing anxiety

The second language writing anxiety inventory (SLWAI)^31^ was utilized to measure writing anxiety. Cheng^31^ affirmed the reliability and validity of this scale. The participants were asked to answer three dimensions with 21 items in a 5-point Likert scale. Somatic anxiety dimension comprised of 7 items that referred to the physiological effects of anxiety (e.g., I feel my heart pounding when I write English compositions under time constraint). Cognitive anxiety dimension included 8 items that pertained to the mental aspects of anxiety (e.g., While writing English compositions, I feel worried and uneasy if I know they will be evaluated). Avoidance behavior dimension was composed of 6 items that indicated a tendency to avoid completing writing assignments or even retreating from such tasks altogether (e.g., I usually do my best to avoid writing English compositions). These dimensions have high internal consistency, with respective values of 0.763, 0.720, and 0.682, being higher or close to the accepted threshold of 0.7.

#### Writing difficulty

To evaluate the difficulty level of writing for students, we have utilized a collection of 12 items (e.g., I can’t write appropriate English sentences to express my ideas) from Wu^54^. These items are evaluated using a 5-point Likert scale from 1 “strongly disagree” to 5 “strongly agree”. The instrument as a whole exhibited a reliability coefficient of 0.873, which surpasses the accepted threshold of 0.7.

#### Data collection

Informed consent was obtained from all participants involved in the study. Data was collected from participants of the 2022 FLTRP∙ETIC Cup English Writing Contest at a university in Eastern China. To evaluate the EFL writing performance of the participants, writing scores were used. These scores were sourced primarily from the official results released by the organizers of the contest. This approach ensures that the evaluation of each participant’s writing skills is based on a standardized and authoritative assessment, reflecting their actual performance in the competition. After the writing contest, participants were asked to complete a questionnaire, which measured self-regulated writing strategies, writing anxiety, and writing difficulty. They were also informed that their data would be kept confidential and used solely for research purposes, and were free to withdraw from the study at any time.

#### Data analysis

This study utilized fsQCA, a method that is suitable for exploring complex configurations of constructs^16^. fsQCA involves assessing the connections between the outcome variable (i.e., writing performance) and all possible combinations of binary states (i.e., presence or absence of its causal conditions). The software fsQCA 3.0 was used.

fsQCA entails a pre-data analysis calibration process^16^. This study utilized the direct method, employing a three-value scheme, to calibrate both causal conditions and outcome measures, which is consistent with previous research^55^^,^^56^. The three-value scheme prescribes the identification of three anchors for every set, encompassing the threshold for full membership, the threshold for full non-membership, and the cross-over point^16^. The calibration procedure then utilizes a logistic function to allocate values to these anchors, leading to the conversion of outcomes and causal conditions into fuzzy membership scores on the log odds of full membership by means of the fsQCA3.0 software^57^. This study used results and antecedent of 95%, 50%, and 5% quantile values. In addition, to limit “researcher degrees of freedom” and avoid “distortion of the results”, we applied the same calibration rule—the 95th, 50th, and 5th percentiles—consistently across all outcomes and causal conditions in this study^58^. Table 1 summarizes the calibration thresholds of the fuzzy sets.

**Table 1:** Calibration of set membership.

Construct	Thresholds		
	Full membership (95th percentile)	Cross-over point (50th percentile)	Full non-membership (5th percentile)
WP	85.00	74.00	54.65
CS	6.67	5.21	3.30
MS	6.70	5.00	2.97
SBS	6.89	4.65	3.00
MRS	6.83	5.37	3.56
WA	3.69	2.78	1.64
WD	4.06	2.83	1.50

*WP* writing performance, *CS* cognitive strategies, *MS* metacognitive strategies, *SBS* social-behavioral strategies, *MRS* motivational regulation strategies, *WA* writing anxiety, *WD* writing difficulty.

Then based on calibrated fuzzy sets, we conducted a necessary condition analysis and a sufficient condition analysis. The results would be analyzed in the following section.

### Ethics declarations

All methods were carried out in accordance with relevant guidelines and regulations. This study was carried out in accordance with the recommendations of the Ethics Committee of Qufu Normal University. Informed consent was obtained from all subjects.

## Results

### Descriptive statistics

Table 2 provided descriptive statistics of participants’ writing performance, self-regulated writing strategies, writing anxiety, and perceived writing difficulty. On average, the writing performance scored 72.24, indicating a relatively high level. However, there was a large range in scores, with the maximum being 90 and the minimum being 29. The most commonly used self-regulated writing strategies were motivational regulation strategies, followed by cognitive, metacognitive, and social-behavioral strategies. The mean scores for writing anxiety and difficulty were 2.75 and 2.81 respectively, and the highest and lowest scores for each were 5 and 1.10, and 5 and 1.17, respectively. According to West et al.^59^, skewness values less than |2| and kurtosis values less than |7| indicate a lack of significant deviation from normality. The skewness and kurtosis presented in Table 2 confirm the absence of any notable departure from normality.

**Table 2:** Descriptive statistics of writing performance, writing strategies, writing anxiety, and writing difficulty (n = 94).

Construct	Mean	SD	Skewness	Kurtosis
Writing performance	72.24	10.77	− 1.86	4.66
Cognitive strategies	5.17	1.04	− 0.29	− 0.59
Metacognitive strategies	4.95	1.11	− 0.35	− 0.33
Social-behavioral strategies	4.72	1.15	0.19	− 0.62
Motivational regulation strategies	5.32	1.07	− 0.55	− 0.01
Writing anxiety	2.75	0.62	0.42	0.68
Writing difficulty	2.81	0.77	− 0.18	0.62

### Analysis of necessary conditions

Crucial to consider are necessary conditions that play a vital role in determining the outcome as their presence is an indispensable element^60^. To put it simply, without the existence of a necessary condition, the outcome is impossible to realize^61^^,^^62^. Employing the software of fsQCA 3.0, Table 3 illustrates an inquiry into necessary conditions for both high and low writing performance.

**Table 3:** Analysis of necessary conditions for high and low writing performance.

Conditions tested	High writing performance		Low writing performance	
	Consistency	Coverage	Consistency	Coverage
CS	0.720	0.732	0.624	0.564
~ CS	0.572	0.631	0.704	0.691
MS	0.702	0.724	0.663	0.608
~ MS	0.620	0.674	0.699	0.676
SBS	0.668	0.732	0.616	0.600
~ SBS	0.635	0.650	0.725	0.660
MRS	0.710	0.721	0.640	0.578
~ MRS	0.584	0.646	0.690	0.679
WA	0.650	0.672	0.721	0.663
~ WA	0.674	0.731	0.643	0.620
WD	0.611	0.662	0.726	0.700
~ WD	0.724	0.748	0.650	0.597

The symbol (~) indicates the absence of condition.

*CS* cognitive strategies, *MS* metacognitive strategies, *SBS* social-behavioral strategies, *MRS* motivational regulation strategies, *WA* writing anxiety, *WD* writing difficulty.

The presence of a necessary condition is contingent upon meeting the consistency and coverage criteria of at least 0.90 and 0.50, respectively, as stipulated by Ragin^63^ and Pappas et al.^64^. As Table 3 demonstrated, none of the values met this threshold, indicating that there were no independent necessary conditions that account for high writing performance. Similarly, no single variable could be identified as a necessary condition for low writing performance. Thus, there were no necessary conditions to produce the outcome of high or low writing performance. The outcome required a combination of conditions, implying that multiple conditions should be integrated for configuration analysis.

### Analysis of sufficient conditions

While a necessary condition is always a prerequisite for an outcome, a sufficient condition denotes that a particular condition or a combination of conditions is capable of leading to the outcome on its own^61,62^.

In order to determine the sufficient conditions for high and low writing performance, the calibrated data was integrated into a fuzzy set truth table and analyzed using fsQCA 3.0 software. The truth table encompassed all possible configurations of the conditions, with the elimination of rows containing less than 2 cases to refine the results. Moreover, configurations with a consistency of less than 0.90 and PRI (Proportional Reduction in Inconsistency) of less than 0.50 were assigned a value of “0” to ensure the sufficiency of the configurations with satisfactory quality^16^^,^^65^. In order for a given configuration to meet the criteria of being “sufficient”, it must possess consistency and coverage values that are ≥ 0.75 and ≥ 0.20^64^^,^^66^. Table 4 illustrates the sufficient solutions for modeling high and low writing performance in a diagrammatic form.

**Table 4:**
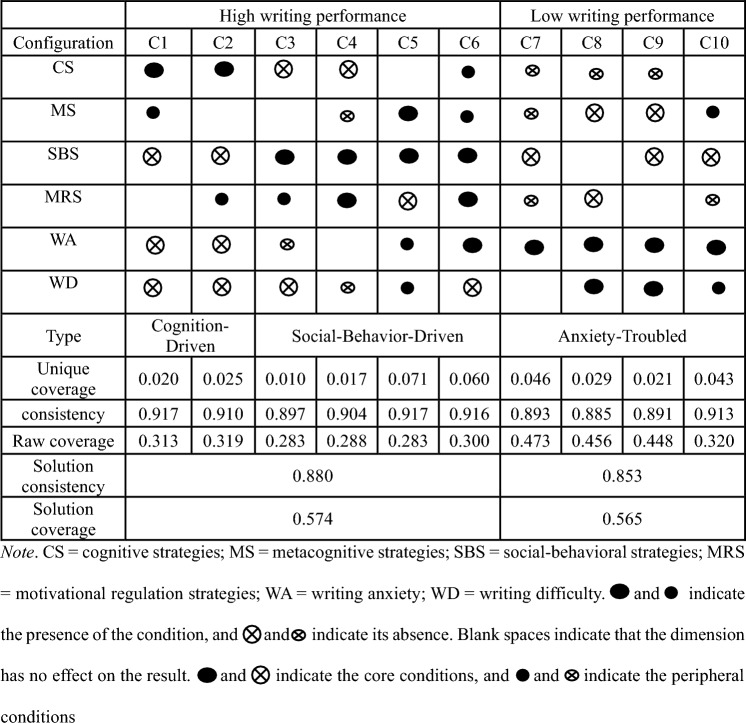
The diagrammatic representation of sufficient solutions of writing performance.

Table 4 provides a visual representation of the conditions sufficient for the outcome. The presence of a condition implies that a learner has a membership score above 0.5, as determined by the calibration procedure. In simpler terms, if a condition is present, it means that the variable value is higher than the median for the sample group. Conversely, if a condition is absent, it means that the variable value is lower than the median for the sample group. Blank spaces indicate that the conditions are not necessary for achieving the desired outcome. This information is based on the research conducted by Misangyi and Acharya^67^ and Bedford et al.^68^.

Grasping configurational solutions gives a complete view of the correlation between the adoption of self-regulated writing strategies and the diverse feelings of writing anxiety and difficulty, both of which can notably affect the writing performance of EFL learners. Our configurational analysis operates on the premise that self-regulated writing strategies, as well as perceptions of writing difficulty and writing anxieties, do not operate independently of each other in influencing learners’ writing performance. Table 4 outlines the 10 configurations that resulted in either high or low writing performance. These configurations serve as evidence that there exist diverse strategic pathways that culminate in equifinal outcomes. This, in turn, corroborates the presence of numerous causal associations in the realm of writing performance. The solution coverages for high writing performance and low writing performance were 0.574 and 0.565, respectively. This indicated a high degree of explanatory power, and all configurations exhibited exceptional levels of consistency, with values of 0.880 and 0.853 in high and low writing performance, respectively. These findings suggested that the configurations were highly effective in producing the desired outcomes.

### Configurations for high writing performance

It is noteworthy that six different configurations (C1–C6) have been identified as potential causal connections that lead to high writing performance (Table 4). The first two configurations (C1–C2) share common core conditions, which involve the implementation of cognitive strategies and low levels of writing anxiety and difficulty. This suggests that cognitive strategies are the primary factor influencing high writing performance in individuals with low levels of writing anxiety and difficult. Consequently, these configurations are categorized as a cognition-driven type, where writing performance is heavily dependent on cognitive writing strategies. An in-depth explanation of these two configurations is provided.

C1: CS*MS* ~ SBS* ~ WA * ~ WD (~ , negation (NOT); *, logical conjunction (AND)) (Table 4). C1 is a configuration that can lead to high writing performance, comprised of a core condition of cognitive strategies, a peripheral condition of metacognitive strategies, and an absence of social-behavioral strategies, writing anxiety, and writing difficulty. This configuration has a unique coverage rate of 0.020 and a consistency measure of 0.917, and it covers 31.3% of sets. This finding suggests that learners with low levels of writing anxiety and difficult can improve their writing ability through the use of cognitive and metacognitive strategies, even if social-behavioral strategies are not used extensively.

C2: CS* ~ SBS*MRS* ~ WA* ~ WD (Table 4). Configuration C2 has the potential to lead to high writing performance. It consists of a core condition of cognitive strategies, a peripheral condition of motivational regulation strategies, and an absence of social-behavioral strategies, writing anxiety, and writing difficulty. This configuration has a unique coverage rate of 0.025 and a consistency measure of 0.910, covering 31.9% of sets. It implies that learners with minimal writing anxiety and difficulty can enhance their writing performance by utilizing more cognitive and motivational regulation strategies, even if they use social-behavioral strategies less frequently.

Configurations C3–C6 are categorized as a social-behavior-driven type due to their shared core conditions centered on the incorporation of social-behavioral strategies, indicating that the adoption of these strategies is the most important factor for high writing performance. These four configurations are further explained in detail.

C3: ~ CS*SBS*MRS* ~ WA* ~ WD (Table 4). Configuration C3 suggests that high writing performance can be achieved without the presence of writing difficulty as core conditions and writing anxiety as peripheral conditions, but with the presence of social-behavioral and motivational strategies as core and peripheral conditions, respectively. This configuration has a unique coverage rate of 0.010, a consistency of 0.897, and covers 28.3% of sets. Therefore, it is suggested that individuals who are not troubled with writing anxiety and difficult may benefit from an increased usage of social-behavioral and motivational strategies to improve their writing performance, even if cognitive strategies are not relied upon as heavily.

C4: ~ CS* ~ MS*SBS*MRS* ~ WD (Table 4). Configuration C4 shows that high writing performance can be achieved with the absence of writing difficulty as a peripheral condition, and the presence of social-behavioral strategies and motivational regulation strategies as core conditions, cognitive strategies as a core condition’s absence, and metacognitive strategies as a peripheral condition’s absence. The unique coverage rate is 0.017, the consistency is 0.904, and the results cover 28.8% of sets. This indicates that when the perceived writing difficulty is low, a higher level of use of social-behavioral strategies and motivational strategies can improve learners’ writing performance, even when the use of cognitive strategies and metacognitive strategies is low.

C5: MS*SBS* ~ MRS*WA*WD (Table 4). Configuration C5 is the most explainable configuration of high writing performance, with the presence of metacognitive strategies and social-behavioral strategies, and the absence of motivational strategies as core conditions, the presence of perceived writing anxiety and difficulty as peripheral conditions. It boasts a high level of consistency (0.917) and unique coverage (0.071), and covers 28.3% of sets, which is indicative of its ability to explain a significant proportion of the results that lead to successful writing. Despite the challenges posed by perceived writing anxiety and difficult, learners can still strive to improve their writing performance by employing a greater number of metacognitive and social-behavioral strategies.

C6: CS*MS*SBS*MRS*WA * ~ WD (Table 4). Configuration C6 reveals a coverage rate of 0.060 and a consistency of 0.916, covering 30.0% of sets. This configuration consists of the absence of writing difficulty and the presence of writing anxiety as core conditions, high writing performance can be achieved by using social-behavioral strategies and motivational strategies as core conditions, and cognitive strategies and metacognitive strategies as a peripheral condition. It is possible for learners to achieve a high level of writing performance even if they experience a high degree of writing anxiety, as demonstrated by the evidence from C6. This can be accomplished through the implementation of cognitive, metacognitive, social-behavioral, and motivational strategies.

### Configurations for low writing performance

Table 4 demonstrates that four distinct configurations, C7–C10, exhibit low writing performance. These configurations share a core condition of high writing anxiety, as well as a lack of self-regulated writing strategies. This suggests that the primary cause of the low writing performance is the presence of heightened writing anxiety and the absence of certain writing strategies. Consequently, these four configurations can be classified as an anxiety-troubled type. Further details of these configurations are provided.

C7: ~ CS* ~ MS* ~ SBS* ~ MRS *WA (Table 4). C7 is a configuration with higher levels of writing anxiety, lower level of use of cognitive strategies, metacognitive strategies, social-behavioral strategies, and motivational strategies. The unique coverage is 0.046, the consistency is 0.893, and it covers 47.3% of sets. This indicates that such a configuration can result in low writing performance among learners.

C8: ~ CS* ~ MS* ~ MRS *WA*WD (Table 4). C8 indicates that a diminished use of cognitive, metacognitive, and motivational strategies can negatively impact writing performance in learners who experience high levels of writing anxiety and difficult. This conclusion is supported by a unique coverage of 0.029 and a consistency of 0.885. And it covers 45.6% of sets.

C9: ~ CS* ~ MS* ~ SBS* WA*WD (Table 4). C9 highlights that when dealing with learners who possess a high level of writing anxiety and difficult, a low usage of cognitive, metacognitive, and social-behavioral strategies may result in poor writing performance. The unique coverage rate is recorded at 0.021, the consistency stands at 0.891, and it covers 44.8% of sets.

C10: MS* ~ SBS* ~ MRS *WA*WD (Table 4). C10 shows that in the context of individuals who exhibit high level of perceived writing anxiety and difficulty, a reduced employment of social-behavioral and motivational strategies can result in low writing performance, despite a high level of utilization of metacognitive strategies. This is indicated by a unique coverage rate of 0.043 and a consistency score of 0.913. And it covers 32.0% of sets.

## Discussion and implications

Table 4 presents configurations contingent upon the attributes of EFL learners, including their utilization of self-regulated writing strategies, writing anxieties, and perception of writing difficulties. These elements have significant impacts on writing performance, with configurations C1–C6 indicating high writing performance, and configurations C7–C10 indicating low writing performance. Three general types of configurations were identified: cognition-driven, social-behavior-driven, and anxiety-troubled.

If EFL learners possess a high level of cognitive and metacognitive strategies, along with low levels of writing anxiety and difficult, as per configuration C1, they are likely to achieve high writing performance. The employment of social-behavior strategies is not crucial for achieving such performance, and the presence of motivational strategies does not significantly affect the outcome. According to configuration C2, EFL learners can attain high writing performance if they possess a wealth of cognitive and motivational strategies, and concurrently experience a low degree of perceived writing anxiety and difficulty. The possession of social-behavioral strategies is not a fundamental requirement for high writing performance, and metacognitive strategies are inconsequential.

The first two configurations (C1–C2) share common core conditions, which involve the implementation of cognitive strategies and low levels of writing anxiety and difficulty, which are categorized as the cognition-driven type, where writing performance is heavily dependent on cognitive writing strategies. This type partially aligns with Teng and Zhang^8^, which indicate that while motivational regulation strategies directly and indirectly affect EFL students’ writing performance and correlate significantly with their use of cognitive, metacognitive, and social behavior strategies, only cognitive and metacognitive strategies were significant mediators.

C3 shows that if EFL learners do not experience high level of writing anxiety or writing difficulty, it is probable that they can achieve high writing performance by utilizing cognitive strategies and metacognitive strategies, in addition to social-behavioral strategies and motivational strategies. C4 suggests that high writing performance can be realized by mitigating the writing difficulty and implementing social-behavioral and motivational writing strategies, even if cognitive and metacognitive strategies are not employed. In accordance with C5, it is possible for EFL learners to achieve high writing performance despite lacking in cognitive strategies and regardless of their possession of metacognitive strategies. This can be achieved through the possession of more social-behavioral strategies and motivational strategies, coupled with low levels of perceived writing anxiety and difficulty. According to the configuration C6, it is possible for EFL learners to achieve high levels of writing performance despite lacking in cognitive strategies and metacognitive strategies, provided that they possess an abundance of social-behavioral strategies and motivational strategies, and also maintain low levels of perceived writing difficulty. Furthermore, this outcome is not influenced by their level of writing anxiety.

Configurations C3–C6 are categorized as a social-behavior-driven type due to their shared core conditions centered on the incorporation of social-behavioral strategies. This finding aligns with the work Mohseniasl^69^, who highlights the role of explicit writing strategy instruction in alleviating writing difficulties, supporting our observation that focusing on specific types of writing strategies, such as social-behavioral and motivational, can lead to improved writing outcomes.

In the case of low writing performance, as per C7, EFL learners who exhibit high levels of writing anxiety and perceived writing difficulty are likely to experience a decline in writing performance if they lack cognitive strategies and metacognitive strategies and social-behavioral strategies, regardless of their possession of additional motivational strategies. As per C8, it is evident that EFL learners who possess high writing anxiety and perceived writing difficulty may face challenges in achieving high writing performance if they lack sufficient social-behavioral strategies and motivational strategies. This is irrespective of whether they possess more metacognitive strategies and regardless of whether they have more cognitive strategies or not. C9 posits that in the event that EFL learners exhibit high levels of anxiety when writing, their writing performance may suffer if they lack social-behavioral strategies and cognitive strategies, metacognitive strategies, and motivational strategies. Whether or not they experience significant writing difficulty is of no consequence. C10 posits that EFL learners who exhibit high levels of writing anxiety and perceived writing difficulty, may experience diminished writing performance if they do not possess the necessary cognitive strategies as well as metacognitive strategies and motivational strategies. Notably, the presence of social-behavioral strategies is not a key determinant of this outcome.

C7-C10, exhibit low writing performance. These configurations share a core condition of high writing anxiety and can be classified as an anxiety-troubled type. This finding is partially in line with Khosravi et al.^70^, who identified a significant negative relationship between writing anxiety and EFL learners’ writing performance, emphasizing the detrimental impact of high anxiety levels on writing.

This study provides valuable insights into the various self-regulated writing strategies that EFL learners can adopt to improve their L2 writing performance, depending on their degree of writing anxiety and difficult. The results are of great significance to the field of pedagogy, as they demonstrate the configurational impacts of writing strategies, writing anxiety, and writing difficulty on writing performance. Consequently, instructors of EFL writing classes can introduce tailored interventions to enhance learners’ writing performance. Additionally, this study proposes an alternative approach to promote the use of writing strategies, taking into account individual characteristics such as perceptions of writing anxiety and writing difficulty.

## Conclusion

This study presents a novel approach to evaluating the potential impact of self-regulated writing strategies on writing performance of Chinese EFL learners. The research acknowledges influence of self-regulated writing strategies, writing anxiety and perceived writing difficulty on writing performance. To evaluate the role of these factors and their collective impact, a fuzzy set qualitative comparative analysis (fsQCA) is used. The results indicate that diverse configurations can lead to either high or low writing performance. Specifically, two configurations fall under the cognition-driven type, which highlights the importance of cognitive writing strategies in high writing performance, while four configurations of the social-behavior-driven type emphasize the significance of social-behavioral writing strategies in high writing performance. On the other hand, it can be inferred that a lack of writing strategies coupled with writing anxiety may result in low writing performance, as exemplified by the four instances of the anxiety-troubled configuration. The above configurations provide educators and instructors with valuable insights on how to provide tailored guidance or corrective measures that can enhance writing performance of EFL learners, depending on the particular configuration, which includes both core and peripheral conditions.

This study provides valuable insights; however, its limitations cannot be ignored. To begin with, the participants were selected in one university, thus, the results cannot be generalized to a wider population. Instead, they can contribute to a better comprehension of the intricate relationships between self-regulated writing strategies, writing anxiety, writing difficulty, and writing performance. Additionally, most data in this study were collected from self-report questionnaires. It’s important to recognize that self-reports, while insightful, can be subject to social desirability bias. This occurs when respondents modify their answers to align with perceived social expectations, potentially skewing the results. To enhance the validity of future research, a more diverse methodological approach is recommended. Integrating objective assessment tools, such as direct behavioral observations or technology-assisted data collection like keystroke analysis, could complement self-reported data. These methods would not only offset the limitations of self-reports but also provide a richer, deeper understanding of the writing process in L2 learners. Lastly, it is essential to note that this study only focused on writing strategies, writing anxiety, and writing difficulty, while writing is a multifaceted and complex process that involves a range of other factors that could affect writing performance. Future research endeavors should consider a broader range of variables to examine the reasons for the variation in writing performance with different combinations of conditions.

## Data Availability

The datasets used and/or analyzed during the current study are available from the corresponding author on reasonable request.

## Appendix 1: Brief introduction of FLTRP·ETIC Cup English Writing Contest

The FLTRP·ETIC Cup English Writing Contest, initiated in 2013, has become a significant event in the field of English language teaching in China. The contest’s rigorously organized and executed processes ensure objective and fair evaluation, affirming its standardization and effectiveness. The contest’s topics and evaluation criteria, designed by a team of professionals, aim to comprehensively assess students’ English writing skills. More information about the FLTRP·ETIC Cup English Writing Contest can be found at https://uchallenge.unipus.cn/.

## Appendix 2: Brief introduction of 2022 FLTRP·ETIC Cup English Writing Contest

The preliminary content of the 2022 FLTRP·ETIC Cup English Writing Contest requires writing one argumentative essay (about 500 words) and one explanatory essay (300–500 words), with a total writing time of 120 min and a full score of 100. The argumentative essay, titled Big Data and Freedom of Choice, guides participants to discuss whether massive information truly provides abundant choices for people’s lives. The expository essay requires participants to choose one of the twenty-four solar terms, introducing its name, meaning, related customs, and  so on.

## Appendix 3: Scoring scheme of writing tasks

### Argumentative writing

I. Content/Ideas (40%)
  1. Writing effectively addresses the topic and the task.
  2. Writing presents an insightful position on the issue.
  3. The position is strongly and substantially supported or argued.
II. Organization/Development (30%)
  1. Writing is well-organized and well-developed, using appropriate rhetorical devices (e.g. exemplifications, classification, analysis, comparison/contrast, etc.) to support the thesis or to illustrate ideas.
  2. Writing displays coherence, progression, consistency and unity.
  3. Textual elements are well-connected through explicit logical and/or linguistic transitions.
III. Language (30%)
  1. Spelling is accurate.
  2. Writing displays consistent facility in use of language.
  3. Writing demonstrates appropriate register, syntactic variety, and effective use of vocabulary.

### Expository writing

I. Content/Ideas (40%)
  1. Writing effectively addresses the topic and the task.
  2. Writing presents a clear thesis.
  3. Writing maintains a formal style and an objective tone.
II. Organization/Development (30%)
  1. Writing is well-organized and well-developed, using appropriate development patterns (e.g., definition, illustration, casual analysis, process analysis, classification, comparison/contrast, etc.) to support the thesis or to illustrate ideas.
  2. Writing displays coherence, progression, consistency and unity.
  3. Textual elements are well-connected through explicit logical and/or linguistic transitions.
III. Language (30%)
  1. Spelling is accurate.
  2. Writing displays consistent facility in use of language.
  3. Writing demonstrates appropriate register, syntactic variety, and effective use of vocabulary.

## References

[1] Santangelo T, Harris KR, Graham S (2007). Self-regulated strategy development: A validated model to support students who struggle with writing. Learn. Disabil. Contemp. J..

[2] Wengelin Å, Sullivan KH, Lindgren E (2006). Examining pauses in writing: Theory, methods and empirical data. Computer Keystroke Logging and Writing.

[3] Shen B, Bai B, Xue W (2020). The effects of peer assessment on learner autonomy: An empirical study in a Chinese college English writing class. Stud. Educ. Eval..

[4] Teng LS (2016). Changes in teachers’ beliefs after a professional development project for teaching writing: Two Chinese cases. J. Educ. Teaching..

[5] Pintrich PR (2000). Multiple goals, multiple pathways: The role of goal orientation in learning and achievement. J. Educ. Psychol..

[6] Zimmerman BJ, Kitsantas A (2002). Acquiring writing revision and self-regulatory skill through observation and emulation. J. Educ. Psychol..

[7] Bai B, Wang J, Nie Y (2021). Self-efficacy, task values and growth mindset: What has the most predictive power for primary school students’ self-regulated learning in English writing and writing competence in an Asian confucian cultural context?. Camb. J. Educ..

[8] Teng LS, Zhang LJ (2016). Fostering strategic learning: The development and validation of the writing strategies for motivational regulation questionnaire (WSMRQ). Asia Pac. Educ. Res..

[9] De Silva R, Graham S (2015). The effects of strategy instruction on writing strategy use for students of different proficiency levels. System.

[10] Bai B, Guo W (2018). Influences of self-regulated learning strategy use on self-efficacy in primary school students’ English writing in Hong Kong. Read. Writ. Q..

[11] Bloom LZ, Rose M (1985). Anxious writers in context: Graduate school and beyond. When a Writer Can’t Write.

[12] Rasool U, Qian J, Aslam MZ (2023). An investigation of foreign language writing anxiety and its reasons among pre-service EFL teachers in Pakistan. Front Psychol..

[13] Hassan, B. A. *The Relationship of Writing Apprehension and Self-Esteem to the Writing Quality and Quantity of EFL University Students*. https://files.eric.ed.gov/fulltext/ED459671.pdf (2001).

[14] Cho M (2018). Task complexity and modality: Exploring learners’ experience from the perspective of flow. Mod. Lang. J..

[15] Sasayama S (2016). Is a ‘complex’ task really complex? Validating the assumption of cognitive task complexity. Mod. Lang. J..

[16] Ragin CC (2008). Redesigning Social Inquiry: Fuzzy Sets and Beyond.

[17] Hertel S, Karlen Y (2021). Implicit theories of self-regulated learning: Interplay with students’ achievement goals, learning strategies, and metacognition. Brit. J. Educ. Psychol..

[18] Wirth J, Stebner F, Trypke M, Schuster C, Leutner D (2020). An interactive layers model of self-regulated learning and cognitive load. Educ. Psychol. Rev..

[19] Teng MF, Huang J (2019). Predictive effects of writing strategies for self-regulated learning on secondary school learners’ EFL writing proficiency. Tesol Quart..

[20] Bai B, Guo W (2021). Motivation and self-regulated strategy use: Relationships to primary school students’ english writing in Hong Kong. Lang. Teach. Res..

[21] Teng MF, Qin C, Wang C (2022). Validation of metacognitive academic writing strategies and the predictive effects on academic writing performance in a foreign language context. Metacogn. Learn..

[22] Bai R, Hu G, Gu PY (2014). The relationship between use of writing strategies and english proficiency in Singapore primary schools. Asia Pac. Educ. Res..

[23] Teng MF (2019). A comparison of text structure and self-regulated strategy instruction for elementary school students’ writing. Engl. Teach. Pract. Crit..

[24] Zhang LJ, Qin TL, Haukås Å, Bjǿrke C, Dypedahl M (2018). Validating a questionnaire on EFL writers’ metacognitive awareness of writing strategies in multimedia environments. Metacognition in Language Learning and Teaching.

[25] Teng MF, Wang C, Zhang LJ (2022). Assessing self-regulatory writing strategies and their predictive effects on young EFL learners’ writing performance. Assess. Writ..

[26] Harris KR, Santangelo T, Graham S (2008). Self-regulated strategy development in writing: Going beyond NLEs to a more balanced approach. Instr. Sci..

[27] Harris KR, Graham S, MacArthur C, Reid R, Mason LH, Zimmerman BJ, Schunk DH (2021). Self-regulated learning processes and children’s writing. Handbook of Self-Regulation of Learning and Performance.

[28] Bazerman C, MacArthur CA, Graham S, Fitzgerald J (2016). What do sociocultural studies of writing tell us about learning to write. Handbook of Writing Research.

[29] Zimmerman BJ (2002). Becoming a self-regulated learner: An overview. Theory Pract..

[30] Qin LT, Zhang LJ (2019). English as a foreign language writers’ metacognitive strategy knowledge of writing and their writing performance in multimedia environments. J. Writ. Res..

[31] Cheng YS (2004). A measure of second language writing anxiety: Scale development and preliminary validation. J. Second Lang. Writ..

[32] Pae TI (2013). Skill-based L2 anxieties revisited: Their intra-relations and the inter-relations with general foreign language anxiety. Appl. Linguist..

[33] Daly JA, Miller MD (1975). The empirical development of an instrument to measure writing apprehension. Res. Teach. Engl..

[34] Zabihi R (2018). The role of cognitive and affective factors in measures of L2 writing. Writ. Commun..

[35] Zabihi R, Mousavi SH, Salehian A (2018). The differential role of domain-specific anxiety in learners’ narrative and argumentative L2 written task performances. Curr. Psychol..

[36] Abolhasani H, Golparvar SE, Robatjazi MA (2022). Modelling the role of L2 writing anxiety in graph-based composing performance and strategy use. J. Psycholinguist. Res..

[37] Abdel Latif MM (2015). Sources of L2 writing apprehension: A study of Egyptian university students. J. Res. Read..

[38] Lee SY (2005). Facilitating and inhibiting factors in English as a foreign language writing performance: A model testing with structural equation modeling. Lang. Learn..

[39] Payant C, McDonough K, Uludag P, Lindberg R (2019). Predicting integrated writing task performance: Source comprehension, prewriting planning, and individual differences. J. Engl. Acad. Purp..

[40] Pallotti G (2019). Assessing tasks: The case of interactional difficulty. Appl. Linguist..

[41] Rabab’ah G (2003). Communicating problems facing Arab learners of english. J. Lang. Learn..

[42] Al-Khasawneh F (2010). Writing for academic purposes: Problems faced by Arab postgraduate students of the college of business UUM. ESL World.

[43] Rahmat NH, Arepin M, Yunos DRM, Rahaman SASA (2017). Analyzing perceived writing difficulties through the social cognitive theory. People Int. J. Soc. Sci..

[44] Wei X, Zhang W (2020). Investigating L2 writers’ metacognitive awareness about L1–L2 rhetorical differences. J. Engl. Acad. Purp..

[45] Zhou J, Wang S, Wang J (2022). Investigating high schoolers’ L2 writing anxiety, L2 writing self-efficacy, L2 writing self-regulated strategies, and L2 writing engagement: Relationships and mediator. Front. Psychol..

[46] Mason LH, Harris KR, Graham S (2011). Self-regulated strategy development for students with writing difficulties. Theory Pract..

[47] Mallahi O, Amirian SMR, Zareian GR, Adel SMR (2016). An investigation into the individual differences correlates of Iranian undergraduate EFL learners’ writing competence: A mixed methods approach. Iran. J. Appl. Linguist..

[48] Sazideh K, Mallahi O (2021). How might cognitive factors affect Iranian EFL learners’ response to feedback provided on writing? An individual differences perspective. Int. J. Linguist. Lit. Transl..

[49] Ragin CC (1999). Using qualitative comparative analysis to study causal complexity. Health Serv. Res..

[50] Rihoux B (2006). Qualitative comparative analysis (QCA) and related systematic comparative methods: Recent advances and remaining challenges for social science research. Int. Sociol..

[51] Poorkavoos M, Duan Y, Edwards JS, Ramanathan R (2016). Identifying the configurational paths to innovation in SMEs: A fuzzy-set qualitative comparative analysis. J. Bus. Res..

[52] Hair JF, Black WC, Babin BJ, Anderson RE (2009). Multivariate Data Analysis.

[53] Hair JF, Hult GTM, Ringle CM, Sarstedt M, Danks NP, Ray S, Hair JF, Hult GTM, Ringle CM, Sarstedt M, Danks NP, Ray S (2021). An introduction to structural equation modeling. Partial Least Squares Structural Equation Modeling (PLS-SEM) Using R. Classroom Companion: Business.

[54] Wu SJR (2003). A comparison of Learners’ Beliefs About Writing in Their First and Second Language: Taiwanese Junior College Business-Major Students Studying English.

[55] Campbell J, Sirmon D, Schijven M (2016). Fuzzy logic and the market: A configurational approach to investor perceptions of acquisition announcements. Acad. Manage. J..

[56] Fiss PC (2011). Building better causal theories: A fuzzy set approach to typologies in organization research. Acad. Manag. J..

[57] Witt MA, Fainshmidt S, Aguilera RV (2022). Our board, our rules: Nonconformity to global corporate governance norms. Admin. Sci. Q..

[58] Douglas S, Berthod O, Groenleer M, Nederhand J (2020). Pathways to collaborative performance: Examining the different combinations of conditions under which collaborations are successful. Policy Soc..

[59] West SG, Finch JF, Curran PJ, Hoyle RH (1995). Structural equation models with nonnormal variables: Problems and remedies. Structural Equation Modeling: Concepts, Issues and Applications.

[60] Dul J (2016). Necessary condition analysis (NCA): Logic and methodology of ‘‘necessary but not sufficient’’ causality. Organ. Res. Methods.

[61] Fiss PC (2007). A set-theoretic approach to organizational configurations. Acad. Manag. Rev..

[62] Schneider MR, Eggert A (2014). Embracing complex causality with the QCA method: An invitation. J. Bus. Mark. Manag..

[63] Ragin CC (2006). Set relations in social research: Evaluating their consistency and coverage. Polit. Anal..

[64] Pappas IO, Woodside AG (2021). Fuzzy-set qualitative comparative analysis (fsQCA): Guidelines for research practice in information systems and marketing. Int. J. Inf. Manag..

[65] Mattke J, Maier C, Reis L, Weitzel T (2021). Bitcoin investment: A mixed methods study of investment motivations. Eur. J. Inf. Syst..

[66] Rasoolimanesh SM, Ringle CM, Sarstedt M, Olya H (2021). The combined use of symmetric and asymmetric approaches: Partial least squares-structural equation modeling and fuzzy-set qualitative comparative analysis. Int. J. Contemp. Hosp. Manag..

[67] Misangyi VF, Acharya AG (2014). Substitutes or complements? A configurational examination of corporate governance mechanisms. Acad. Manage. J..

[68] Bedford DS, Malmi T, Sandelin M (2016). Management control effectiveness and strategy: An empirical analysis of packages and systems. Account. Org. Soc..

[69] Mohseniasl F (2014). Examining the effect of strategy instruction on writing apprehension and writing achievement of EFL learners. Theory Pract. Lang. Stud..

[70] Khosravi R, Dastgoshadeh A, Jalilzadeh K (2023). Writing metacognitive strategy-based instruction through flipped classroom: An investigation of writing performance, anxiety, and self-efficacy. Smart Learn. Environ..

